# Correlation between benign joint hypermobility syndrome and primary focal hyperhidrosis in children: a novel concept

**DOI:** 10.1186/s12891-020-03264-8

**Published:** 2020-04-24

**Authors:** Vadood Javadi Parvaneh, Hoda Shahvaladi, Khosro Rahmani, Sara Javdani Yekta, Fatemeh Abdollah Gorji, Reza Shiari, Fahimeh Abdollahimajd

**Affiliations:** 1 Pediatric Pathology Research Center, Shahid Beheshti University of Medical Sciences, Tehran, Iran; 2 Department of Pediatric Rheumatology, Shahid Beheshti University of Medical Sciences, Tehran, Iran; 3 Mofid Children’s Hospital, Shariati Ave, Hosseinieh Ershad, Tehran, Iran; 4 Skin Research Center, Shahid Beheshti University of Medical Sciences, Tehran, Iran; 5 Clinical Research Development Unit, Shohada-e Tajrish Hospital, Shahid Beheshti University of Medical Sciences, Tehran, Iran

**Keywords:** Benign joint hypermobility syndrome (BJHS), Primary focal hyperhidrosis, Pediatrics/children, Palmoplantar hyperhidrosis, Axillary hyperhidrosis

## Abstract

**Background:**

Benign joint hypermobility syndrome (BJHS) is one of the most common hereditary connective tissue disorders in children in which autonomic nervous system involvement has been reported. This study aimed to evaluate the frequency of primary focal hyperhidrosis in children with BJHS.

**Methods:**

This observational-analytical study was conducted in a case-control setting on children aged 3 to 15 years in 2018 at Mofid Children’s Hospital, Tehran, Iran. Benign joint hypermobility syndrome was diagnosed according to the Brighton criteria; then, the patients referred to a dermatologist for evaluation of hyperhidrosis.

**Results:**

In total, 130 eligible patients with confirmed BJHS and 160 age- and sex-matched healthy subjects were enrolled in this study. Primary focal hyperhidrosis (PFH) was seen in 56.2 and 16.3% of the cases and controls, respectively, indicating a significant difference (*P* < 0.05). The severity of hyperhidrosis did not differ between the two groups.

**Conclusion:**

Although the results of the study showed a significant correlation between BJHS and PFH, more comprehensive studies are needed to confirm these findings.

## Background

Generalized joint hypermobility (GJH) defined as increased mobility of joints is diagnosed on the basis of various sets of clinical criteria including Beighton 9-point scoring system and Shiari-Javadi criteria [1–3]. When the patient has signs and symptoms related to GJH, the term joint hypermobility syndrome (JHS) is considered. Benign joint hypermobility syndrome (BJHS) is a benign hereditary connective tissue disorder in which musculoskeletal symptoms present in the absence of other rheumatologic disorders such as Marfan syndrome, Ehlers-Danlos syndrome, and osteogenesis imperfecta [4, 5]. Joint laxity, dislocation and subluxation, musculoskeletal pain, together with arthritis, are its main articular manifestations. In addition, some extra-articular features including psychological problems and the involvement of the skin, peripheral nervous system and urinary system can be mentioned [6–8]. To date, several criteria, such as Brighton, have been proposed for the diagnosis of BJHS. Patients who are suspected of having this syndrome should be examined for all the major and minor criteria, and in case of obtaining the minimum necessary criteria, the diagnosis is confirmed [4, 5]. In previous studies, autonomic nervous system symptoms, generalized anxiety, phobia, and mood disorders have been reported in patients suffering from BJHS [8–10].

On the other hand, hyperhidrosis or excessive sweating affects around 3% of the general population in the United States and it is often categorized as primary and secondary types [11]. Secondary hyperhidrosis (localized or generalized) is correlated with an underlying disorder, such as infections, endocrine, metabolic, neoplastic or neurologic disorders. Primary hyperhidrosis is a localized and symmetric type of excessive sweating that affects usually the palms, soles, axilla or other areas. Primary focal hyperhidrosis (PFH) is not related to any systemic or underlying diseases; however, stress, anxiety and emotional excitement can exacerbate this condition and it has been proposed to arise from over-activity of the sympathetic nervous system [8, 11–13]. Hyperhidrosis may lead to significant psychological and emotional distress and some limitations in social interaction. There are various treatment options available today and its early diagnosis and timely treatment can contribute to improve the quality of life in patients with hyperhidrosis [12, 14].

To the authors’ knowledge, the relationship between hypermobility and hyperhidrosis has not been previously reported in the literature; so, the present study was designed to evaluate the frequency of primary hyperhidrosis in children with BJHS compared to the controls.

## Materials and methods

### Subjects

This observational-analytical study was performed in a case-control setting in the period from May to September 2018 in the outpatient clinics of Mofid Children’ s Hospital, a tertiary referral center, which accepts patients with different ethnicities from all over Iran. The case group included children aged 3 to 15 years with signs and symptoms of BJHS (confirmed by two experienced pediatric rheumatologists). Healthy children aged 3–15 years visiting the outpatient clinic during the same time interval were recruited as the control group; they did not have the criteria for BJHS. Written informed consent was obtained from the parents or guardians.

The subjects were excluded from the study in case of the impossibility of evaluating Beighton and Brighton criteria due to various reasons such as fracture, or joint injury and dislocation, a history of any chronic or underlying diseases, and failure to follow the study course.

### Protocol

Demographic characteristics of all subjects consisting of age, gender, family history of hyperhidrosis, and all other variables were evaluated and determined by interviewing the parents/guardians or studying the patients’ records.

All subjects were initially examined by two pediatric rheumatologists for generalized joint hypermobility based on Beighton score and Shiari-Javadi criteria (Table 1); they were then evaluated for its syndromic nature based on Brighton criteria [4, 15].

**Table 1:** Shiari-Javadi criteria for the diagnosis of generalized joint hypermobility in children. *Adapted from Parvaneh VJ, Shiari R. Proposed modifications to Beighton criteria for the diagnosis of joint hypermobility in children. Indian Journal of Rheumatology. 2016;11(2):97–100*

No.	Maneuvers	Score
**1**	Bilateral passive lateral neck rotation in which nose imaginary line down the frontal appendage of acromioclavicular joint	1
**2**	Passive vertical shoulders hyperextension so that elbows touch together from behind	1
**3**	Passive back hyperextension ≥30°	1
**4**	Bilateral passive elbow hyperextension ≥10°	1
**5**	Bilateral passive 2–5 MCPs hyperextension ≥90°	1
**6**	Thumbs touch volar aspect of forearm passively	1
**7**	Bilateral passive hip internal rotation ≥60°	1
**8**	Bilateral passive knee hyperextension ≥10°	1
**Total score**^**a**^		**8**

^**a**^ The criteria are fulfilled with 6 scores or higher

Exclusion criteria: less than 3 years of age and over 16 years of age, multiple fractures or a history of dislocated optic lens, non- benign musculoskeletal pain, and Marfan or Ehlers-Danlos syndrome

In the next step, the subjects were referred to the dermatology clinic of the same hospital and examined for primary focal hyperhidrosis. In the case of hyperhidrosis, severity, type, and localization were determined and recorded.

For the diagnosis of primary focal hyperhidrosis (PFH), the patients were studied based on the following criteria: a) localized excessive sweating for at least 6 months without underlying diseases, b) at least two of the following: symmetric and bilateral, impairment of daily life activities, at least one episode of excessive sweating per week, age of onset less than 25 years, positive family history, and stops during sleep. Disease severity was determined in the palmoplantar and axillary sites. In the case of volar hyperhidrosis with no visible drops, it was considered as mild; in case of progression towards the fingers or toes, it was moderate; and in cases that the sweat dripped, it was regarded as severe. The hyperhidrosis severity in the axillary site was determined as follows: a sweat stain on the clothes being 5–10, 10–20, and > 20 cm in diameter was considered as mild, moderate, and severe, respectively [16]. The ambient temperature of the indoor clinics was between 23 and 26^°c^.

### Statistical analysis

The collected data were analyzed by the SPSS software, version 25. The presence and severity of hyperhidrosis were compared between the study and control groups. The frequency and percentage were used for qualitative variables, whereas mean, standard deviation, and domain were used for quantitative data. The prevalence of hyperhidrosis was then compared between the two groups with Fisher’s exact test. Its severity and localization were also compared between the two groups by Chi-Square test and logistic regression analysis for predictor variables in the factor of BJHS. The significance level was set at *P* < 0.05.

## Results

This study included 139 cases with confirmed BJHS and 182 control subjects. A total of nine patients and 22 control subjects did not visit the dermatology clinic and were excluded from the study. Therefore, 130 eligible cases and 160 age-and-sex-matched controls were recruited the study. Baseline demographics and clinical features of the two groups are presented in Table 2.

**Table 2:** Demographic Characteristics, Beighton score, and PFH of Subjects

	BJHS No = 130	Controls No = 160	*P*-value	OR	95% CI
Age (year)	8.5 ± 3.2 (3.5–15)	8.5 ± 3.6 (3.5–15)	0.975^*^	–	–
Male	64 (49.2%)	79 (49.4%)	1.0 ^Ɨ^	0.994	0.625–1.580
Positive family history of PFH	41 (31.5%)	30 (18.8%)	0.014 ^Ɨ^	0.501	0.291–.0862
Beighton score	7.33 ± 1.43 (4–9)	0.42 ± 0.63 (0–2)	< 0.001^*^	–	–
PFH	73 (56.2%)	26 (16.3%)	< 0.001 ^Ɨ^	0.152	0.088–0.261
– Palmoplantar	− 73 (56.2%)	− 26 (16.3%)	< 0.001 ^Ɨ^	0.152	0.088–0.261
– Axillary	− 14 (10.8%)	− 8 (5%)	0.076 ^Ɨ^	0.436	0.177–1.074

*Abbreviations*: *BJHS* Benign joint hypermobility syndrome, *No* Number; *PFH* Primary focal hyperhidrosis

^*^Mann-Whitney U

^Ɨ^ Fisher’s Exact Test

Forty-one cases and 30 controls had a positive family history for hyperhidrosis, revealing a significant difference (*P* = 0.014; Table 2). The mean Beighton score was reported as 7.33 ± 1.43 (range: 4–9) and 0.42 ± 0.63 (range: 0–2) in the patient and control groups, respectively, indicating a significant difference (*P* < 0.001; Table 2). Primary focal hyperhidrosis was seen in 56.2 and 16.3% in the patents and healthy subjects, respectively; the difference was statistically significant (*P* < 0.001, OR = 0.152, 95%CI: 0.088–0.261; Table 2). Hyperhidrosis was bilateral in all subjects who had PFH. Moreover, in all cases with hyperhidrosis, palmoplantar hyperhidrosis was present, while 14 (10.8%) patients and 8 (5%) healthy subjects had also axillary hyperhidrosis. No meaningful difference was observed in axillary hyperhidrosis between the two groups (*P* = 0.076; Table 2).

Regarding the severity of hyperhidrosis, 59 (80.8%), 10 (13.7%), and 4 (5.5%) cases reported their PFH as mild, moderate, and severe, respectively. The same information was 23 (88.5%), 3 (11.5%), and 0 (0%) in the control group; no significant difference was observed in PFH severity between the two groups in this respect (*P* = 0.443; Table 3).

**Table 3:** Comparison of severity of PFH in BJHS and control groups

Severity	BJHS	Controls	***P***-value
**PFH**	**No = 73**	**No = 26**	0.443 ^ŧ^
***– Mild***	− 59 (80.8%)	− 23 (88.5%)	
***– Moderate***	− 10 (13.7%)	− 3 (11.5%)	
***– Severe***	− 4 (5.5%)	− 0 (0%)	
**Palmoplantar hyperhidrosis**	**No = 73**	**No = 26**	0.503 ^ŧ^
***– Mild***	− 59 (80.8%)	− 23 (88.5%)	
***– Moderate***	− 11 (15.1%)	− 3 (11.5%)	
***– Severe***	− 3 (4.1%)	− 0 (0%)	
**Axillary hyperhidrosis**	**No = 14**	**No = 8**	0.516 ^ŧ^
***– Mild***	− 10 (71.4%)	− 7 (87.5%)	
***– Moderate***	− 2 (14.3%)	− 1 (12.5%)	
***– Severe***	− 2 (14.3%)	− 0 (0%)	

*Abbreviations*: *BJHS* Benign joint hypermobility syndrome, *No* Number; *PFH* Primary focal hyperhidrosis

^ŧ^ Chi-Square Test

The logistic regression analysis was performed in which BJHS considered as a dependent variable, and the age, gender, PFH, and family history of PFH were selected as independent predictor variables. A total of 290 cases were in the analysis, and the full model was significant (χ^2^ = 54.959, df = 4, *P* < 0.001). This model varies between 17 and 23% of the variance of BJHS. A total of 56.2% of the patients in the BJHS group and 83.8% of the predictions in control group were correct. Coefficients and Wald statistic, degrees of freedom, and probability values were calculated for each of the predictive variables that had a significant effect on the PFH variable (*P* < 0.001, 95%CI: 4.233–15.311); however, the effect of gender (*P* = 0.445, 95%CI: 0.484–1.375), age (*P* = 0.187, 95%CI: 0.976–1.135), and family history of PFH (*P* = 0.439; 95%CI: 0.656–2.639) were not significant (Supplementary Figure 1).

## Discussion

In the present study, the rate of PFH in apparently healthy children was 16.3%. In various published reports, the prevalence of primary hyperhidrosis varies from 2.8% in the United States, to 12.8, 4.6, and 14.5% in Japan, Germany, and Shanghai, China, respectively. Although the exact reason for this discrepancy is not clear, the racial and geographical differences may explain it. Moreover, in recent years, the prevalence of hyperhidrosis has been studied in children and the youth of different societies, revealing a prevalence of 0.5–1.6% [11, 17–20].

To the authors’ knowledge, this is the first study conducted on children to evaluate the association between joint hypermobility and hyperhidrosis. We found that the rate of PFH in patients with BJHS was significantly higher compared to the control subjects and PFH had a significant effect on the prediction of BJHS; however, there was no relationship between the severity of hyperhidrosis and the intensity of hypermobility. Gazit et al. performed a study on 48 patients with JHS and 30 healthy controls to assess the frequency of different complaints related to the autonomic nervous system. They showed that 13% of the cases with JHS and 17% of the healthy subjects suffered from hyperhidrosis; however, there was no significant difference between the two groups [8]. The results of this study contrast our findings; this could be either due to the difference in the sample size of these studies (48 vs. 130) or the difference between the two studied groups in terms of age and ethnicity.

In addition, the study conducted by Gazit et al., showed the majority of patients with JHS suffered from mild to moderate degrees of dysautonomia [8]. The autonomic nervous system has been implicated in several reported extra-articular complaints in patients with JHS such as palpitation, syncope, flushing and dizziness [8, 21]. Several explanations have been suggested for the sympathetic dysregulation related to dysautonomias in these patients including impairment in central sympathetic control, peripheral neuropathy, or deconditioning because of muscle disuse [8].

On the other hand, as previously mentioned, the over-activity of sympathetic nervous system plays a role in primary hyperhidrosis [11]. Taking into account the possible role of autonomic nervous system in both disorders, it would not be surprising to find an association between hyperhidrosis and joint hypermobility; as the results of the present study seems to highlight a correlation between BJHS and PFH. Our findings revealed that PFH in children with BJHS was four times more than in the control group. Therefore, it is proposed that hyperhidrosis may be considered as one of the extra-articular complaints of BJHS related to the autonomic nervous system. However, more comprehensive studies with a larger sample size on different ethnicities, races, and nations, as well as a wider range of ages, including adults are needed to clarify this finding and its exact pathophysiological basis. It is also recommended to evaluate the prevalence of hypermobility in individuals with PFH.

## Conclusion

Taken together, our findings suggested a possible association between hypermobility and PFH in children, which suggests that primary hyperhidrosis as an autonomic nervous system–related symptom, may be an extra-articular manifestation in joint hypermobility.

It is recommended to assess this complaint in patients with hypermobility and refer them to a dermatologist for management and improvement of the quality of life if needed.

## Supplementary information

**Additional file 1: Figure S1.** Observed Groups and Predicted Probabilities

## Data Availability

If requested (please contact vadoodj@gmail.com).

## Abbreviations

BJHS: Benign joint hypermobility syndrome
CI: Confidence interval
DF: Degree of freedom
GJH: Generalized joint hypermobility
JHS: Joint hypermobility syndrome
MCP: Metacarpophalangeal
NO: Number
Yr: Year
OR: Odds ratio
PFH: Primary focal hyperhidrosis
