# Supplementary figures and images for: Correlation between benign joint hypermobility syndrome and primary focal hyperhidrosis in children: a novel concept
Source: BMC Musculoskelet Disord. 2020 Apr 24;21:268. doi: 10.1186/s12891-020-03264-8 (PMC7183110; doi:10.1186/s12891-020-03264-8)

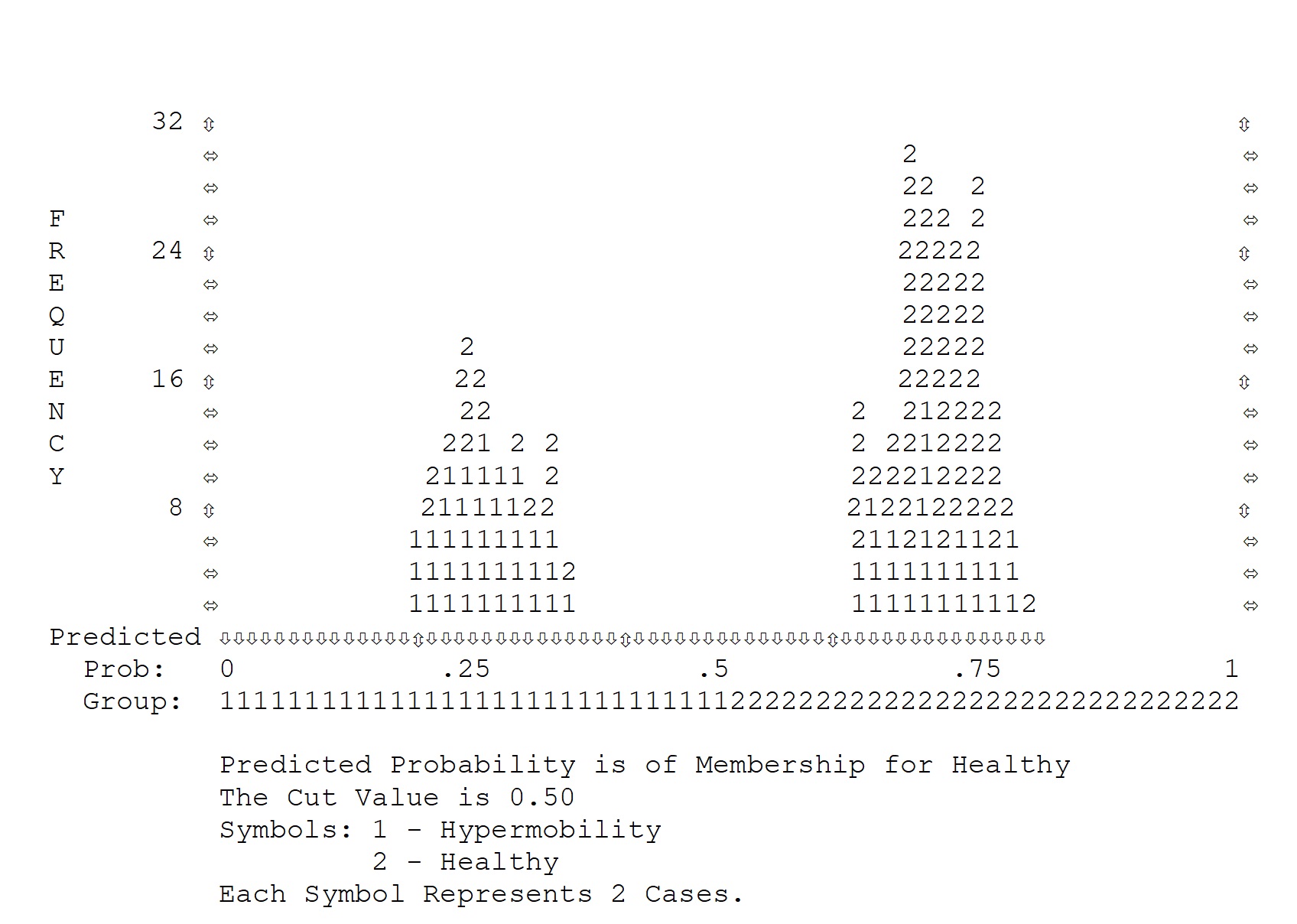

Supplement: Supplementary file 1 — Additional file 1: Figure S1. Observed Groups and Predicted Probabilities [file 12891_2020_3264_MOESM1_ESM.jpg]
